# An Interest-Based Approach for Reducing Network Contentions in Vehicular Transportation Systems

**DOI:** 10.3390/s19102325

**Published:** 2019-05-20

**Authors:** Allan M. de Souza, Guilherme Maia, Torsten Braun, Leandro A. Villas

**Affiliations:** 1 Institute of Computing, University of Campinas, Campinas 13083-852, Brazil; leandro@ic.unicamp.br; 2 Institute of Computer Science, University of Bern, 3012 Bern, Switzerland; 3 Computer Science Department, Federal University of Minas Gerais, Belo Horizonte 31270-901, Brazil; jgmm@dcc.ufmg.br

**Keywords:** traffic management system, floating content, opportunistic content sharing

## Abstract

Traffic management systems (TMS) are the key for dealing with mobility issues. Moreover, 5G and vehicular networking are expected to play an important role in supporting TMSs for providing a smarter, safer and faster transportation. In this way, several infrastructure-based TMSs have been proposed to improve vehicular traffic mobility. However, in massively connected and multi-service smart city scenarios, infrastructure-based systems can experience low delivery ratios and high latency due to packet congestion in backhaul links on ultra-dense cells with high data traffic demand. In this sense, we propose I am not interested in it (IAN3I), an interest-based approach for reducing network contention and even avoid infrastructure dependence in TMS. IAN3I enables a fully-distributed traffic management and an opportunistic content sharing approach in which vehicles are responsible for storing and delivering traffic information only to vehicles interested in it. Simulation results under a realistic scenario have shown that, when compared to state-of-the-art approaches, IAN3I decreases the number of transmitted messages, packet collisions and latency in up to 95%, 98% and 55% respectively while dealing with traffic efficiency properly, not affecting traffic management performance at all.

## 1. Introduction

Recent studies have shown that traffic management systems (TMS) are the basis for dealing with mobility issues [1,2]. TMSs aim to improve the efficiency of the available transportation infrastructure by integrating sensing, processing, and communication technologies to gather and exploit data produced by vehicles, traffic infrastructures, in-road, and roadside sensors, subsystems, and even people to improve vehicular mobility [3,4].

The emergence of advanced communication technologies, such as fifth generation (5G) networks [5] and vehicular networking [6], are paving the way to build TMSs to provide a smarter, faster, and safer transportation. 5G networks will offer TMSs higher bandwidths and improved scalability to support different applications, such as large-scale vehicular congestion control and forecasting, live video sharing and vehicle-to-everything (V2X) communication [7]. Meanwhile, vehicular networking will enable vehicles to produce, share, and process information to meet a more efficient and reliable traffic guidance [6].

There are at least three key issues that need to be addressed for developing a highly efficient TMS to improve traffic efficiency and reliability.

Accurate knowledge about traffic conditions:vehicle re-routing is a fundamental service when dealing with traffic congestion problems [1]. Therefore, knowing the ongoing traffic conditions on roads is crucial. Several TMSs [1,2,8,9] rely on infrastructure-based approaches to build a global traffic view, which might lead to the following limitations: (i) high infrastructure dependence; and (ii) single point of failure. The former might be a bottleneck, due to the high connectivity demands in dense urban areas [10]. Vehicles connected to highly demanded infrastructures might experience low data delivery and packet congestion in backhaul links [11,12]. The latter might motivate cyberattacks at these points, since a malfunctioning component means that an entire area may become unassisted, thus degrading the overall system performance.

Low-latency communication: to meet the strict reliability and scalability requirements, TMS needs to exchange data as efficiently as possible to avoid network contention, provide a high delivery ratio, and induce a low packet collision and low latency on the system. Unfortunately, some TMSs do not consider this issue in their design or just employ a limited hop counting approach, which is not effective [2,9,13,14,15]. On the other hand, some TMSs implement broadcast suppression mechanisms to minimize redundant transmissions while disseminating data through the network [1,8]. However, they spread the data through the entire network rather than forward only to vehicles that have interested in it. Hence, depending on the size of the network, spreading it to all vehicles might be inefficient.

Real-time responsiveness: sudden changes in traffic caused by unexpected incidents might degrade the overall traffic efficiency and also lead to human damages and even deaths, which are extremely serious concerns. Notwithstanding, some TMSs do not employ any mechanism for dealing with unexpected events [1,2,16,17,18]. Others employ mechanisms for dealing with these events by avoiding the traffic congestion in the location of the event and also preventing physical damages [8,14,15]. However, these TMSs neither implement in-network caching nor self-storage mechanisms. Instead, they rely on periodical broadcasts to warn approaching vehicles, thus leading to network contention due to redundant transmissions.

Motivated by the aforementioned issues and the drawbacks of existing TMSs, we propose I am not interested in it (IAN3I), an interest-based approach for improving the network performance in vehicular transportation systems. IAN3I enables vehicles to build an accurate traffic view about a specific area without the support of any infrastructure. Moreover, it delivers such knowledge to vehicles in an opportunistic way according to their interests. Consequently, it does not produce network contentions, which means that IAN3I can deal with vehicular mobility issues efficiently.

The idea of using interest-based messages was first introduced in named-data networking (NDN) [19] to request specific content objects [20]. This approach has shown substantial improvements in network performance when compared to uncontrolled forwarding approaches [21,22]. Therefore, we extended such an idea to IAN3I. However, in AIN3I, the interest-based approach is used to define which vehicle needs to receive the pieces of information about traffic conditions instead of using it to request a specific content. In this way, the key contributions of this work are as follows.

Infrastructure-free traffic sensing: infrastructure-based approaches might lead to unavailability issues since they depend strictly on Roadside Units (RSU) to provide the knowledge about traffic conditions, thus if the RSU becomes unavailable, these systems cannot have any knowledge about the traffic dynamics within the area covered by the RSU [8,13,16]. Therefore, providing a distributed approach (i.e., infrastructure-free) to produce knowledge about traffic conditions in some regions can improve the reliability of the system, once the vehicles themselves can produce such knowledge either during some RSU unavailability or under regions that have no coverage. In this way, IAN3I employs an efficient mechanism to provide a traffic view of specific areas. Some of the design issues considered are: (i) how to build a traffic view from a large area given that vehicles only have knowledge about their limited surroundings? and (ii) how to build such knowledge without inducing network contentions?

Opportunistic content sharing: approaches based on data dissemination algorithms must rebroadcast the traffic information throughout the entire area of interest periodically to ensure that all vehicles interested in it will receive the information. However, such a task might be inefficient depending on the size of the area of interest, the data dissemination algorithm, and the density of vehicles [2,8,14]. Consequently, leading to dissemination of duplicated messages and also unnecessary transmissions (considering that vehicles that have no interest in that information will also receive it), which potentially increase the overhead produced by the system. Therefore, instead of spreading the traffic information of a specific area throughout the entire network, IAN3I holds it close to its relevant area as a floating content object and forwards this content only to vehicles that have interest in it. The floating content is an opportunistic communication paradigm for supporting server-less distributed content sharing [23,24,25] which will be responsible for forwarding the content opportunistically to vehicles that have interest in it and also to ensure its survivability and availability within its relevant area.

In-network data caching: by using the floating content-based approach and the opportunistic content sharing mechanism, IAN3I keeps the knowledge available within a relevant area rather than periodically re-broadcast it throughout the entire network without carrying about the interest of each vehicle, consequently reducing the overhead produced by the system which can lead to network contentions [1,2,8]. In other words, IAN3I provides an efficient mechanism for storing and delivering spatio-temporal information, such as unexpected incident warning and poor traffic conditions.

The rest of this paper is organized as follows: Section 2 provides an overview of the existing TMS approaches. Section 3 introduces IAN3I. The performance evaluation of our proposed approach is described in Section 4. Finally, Section 5 presents conclusions and future work.

## 2. Related Work

Services such as INRIX [26] provide real-time traffic information, which might support drivers to choose routes as fast as possible. In turn, Google Maps and Waze are vehicular navigation systems (VNS) able to forecast traffic congestion and its duration by performing advanced statistical predictive analysis of traffic patterns. Moreover, they also can recommend the fastest routes whenever a route planning is requested.

Furthermore, several TMSs can be found in the literature to improve traffic efficiency and to reduce congestion-related concerns. Most TMSs act reactively, focusing on controlling traffic congestion after its occurrence. In general, these TMSs suggest fastest routes (i.e., routes that avoid congested roads) to vehicles periodically [1,2,13,17]. On the other hand, some TMSs act proactively by identifying unexpected traffic incidents that can produce traffic congestion, thus as soon as such an incident is detected the TMSs suggest alternative routes to vehicles that are going towards the incident, to avoid creating traffic congestion in that location [8,14].

Garip et al. [17] proposed a scalable reactive vehicle-to-vehicle congestion avoidance mechanism, which detects traffic congestion and re-routes vehicles to minimize their trip time. The proposed system is highly scalable, since it is fully-distributed, i.e., it does not rely on any infrastructure to detect congestion and re-route vehicles. Relying on local traffic information, every vehicle estimates the traffic condition within its current road and requests traffic information of alternative paths to surrounding vehicles. In order to minimize computational efforts, vehicles update their routes based on checkpoints rather than computing a global route. Upon receiving the traffic condition from *k*-possible requested routes, it builds a faster route. Finally, to address network contentions, it employs a request and reply technique rather than relying on periodic broadcasts.

Pan et al. [1] introduced DIVERT (Distributed Vehicular Traffic Re-routing System), a hybrid TMS which computes the fastest routes cooperatively to each vehicle based on a global traffic view. The DIVERT architecture is composed of a main server responsible for detecting signs of congestion and a software running on each vehicle, which is responsible for reporting its traffic information (e.g., travel time of each road) to the main server using cellular infrastructure in order to build the traffic view. Moreover, it also employs a vehicular networking based information reporting protocol to minimize network contentions and reduce privacy leakage (e.g., the current location of several vehicles). For improving the traffic efficiency, when traffic congestion is detected, the main server sends a notification message with the global traffic view to the vehicles close to it to enable them to re-route themselves to avoid the congestion. The re-routing algorithm is executed in a cooperative manner to provide a better traffic flow and avoid the bottlenecks in the transportation infrastructure.

Doolan et al. [2] proposed EcoTrec, an eco-friendly TMS, which focuses on reducing carbon emissions while improving traffic efficiency. EcoTrec uses measurements periodically reported by vehicles to a central server to build a global traffic view. Each measurement is reported using an epidemic routing data dissemination protocol to ensure its delivery. Based on the measurements received by each vehicle, the central entity builds the traffic view and then forwards it to vehicles at a predefined interval. Thus, upon receiving a traffic view, vehicles compute the optimal route based on a shortest path algorithm. As a consequence, this reduces carbon emission and improves traffic efficiency, since congested roads have higher fuel consumption rates. It is important to stress that whenever a vehicle receives an updated traffic view, its optimal route is re-calculated.

Wang et al. [14] introduced NRR (Next Road Re-routing), an adaptive next road re-routing TMS for unexpected urban traffic congestion avoidance. NRR saves the cost of obtaining a global traffic view by relying on local information available at RSUs (which are assumed to be deployed at each intersection) to select the best next road for each vehicle. The idea is to avoid the road that contains an unexpected incident rather than computing a whole new route. The local information is built based on vehicles report. In order to detect unexpected congestion, NRR relies on a central server responsible for sending a notification to the RSU closest to the congestion. Thus, the RSU broadcasts such notification to all vehicles within its coverage, enabling them to verify whether their routes go toward some road that will potentially become congested. Thus, the RSU uses the latest traffic information obtained to compute the next road, so vehicles can avoid the unexpected incident based on its local traffic view.

In our previous work, we presented ICARUS (Improvement of traffic Condition through an Alerting and Re-routing System) [8], a TMS to improve traffic condition based on an alerting and re-routing system. It is aware of both the current traffic condition and unexpected traffic incidents. Therefore, when it detects any traffic congestion or an unexpected traffic incident, it creates a warning message and spreads it through the network (based on a predefined area of interest) to warn vehicles about the incident. ICARUS employs a delay-based data dissemination protocol which addresses the broadcast storm problem by relying on a broadcast suppression mechanism based on the sweet spot concept. In addition, to ensure that all vehicles receive the warning, ICARUS periodically rebroadcasts the warning. Finally, to avoid the emergence of congestion, whenever a vehicle receives a warning message, it verifies if it will pass through the congested area or the area with the unexpected traffic incident and requests a new route to a central server that possesses global traffic view.

Grassi et al. [21] introduced Navigo, a location interest-based forwarding mechanism for vehicular NDN. Navigo aims to address frequent connectivity disruptions and sudden network changes in vehicular communications. To do so, instead of forwarding messages to a specific vehicle, it fetches specific pieces of data from other vehicles which potentially have such information. Navigo divides the scenario into smaller regions according to the military grid reference system (MGRS), then it maps each content to a specific cell of the MGRS based on its geographic information and its producer. Also, it provides an efficient algorithm to retrieve pieces of information from the best sources of each region according to a specific interest message.

Ahmed et al. [22] proposed CODIE (Controlled Data and Interest Evaluation), a controlled data and interest evaluation in vehicular NDN. The CODIE’s main idea is to control broadcast storm by restricting the number of hops that an interest message can travel. To do so, CODIE adds a hop-counter to each interest message. When a vehicle receives the interest message, it checks whether it possesses the content requested in the message. If so, the vehicle forwards the content to the vehicle which has requested the content. Otherwise, the vehicle increments the hop-counter and forwards the interest message to another vehicle. This procedure continues until the hop-counter reaches a threshold named as data dissemination limit (DDL).

Table 1 presents a qualitative comparison between some TMSs in the literature and IAN3I. The qualification is based on the following set of criteria: architecture, network challenges addressed, size of traffic view, re-routing algorithms and special features.

As it can be seen, IAN3I differs from existing approaches in three aspects. First, we take full advantage of vehicle-to-vehicle communications to build an accurate traffic view without relying on any additional infrastructure (limitation presented in solutions [1,2,8,13,14]). Thus, vehicles cooperatively share their information with surrounding vehicles to first create a local traffic estimation. Then, using an efficient data dissemination protocol, we select a set of vehicles (ensuring the whole coverage of the area of interest) to spread their local traffic estimations and build a global traffic view. Secondly, it uses an interest-based forwarding mechanism to opportunistically send pieces of information just to vehicles actually interested in it, this approach overcomes the limitation of spreading the content throughout the entire network which is a limitation presented in [1,2,8]. Therefore, it significantly reduces the amount of data disseminated over the network, resulting in a more scalable approach. Thirdly, it employs an in-network caching strategy to store pieces of information within their relevant areas. Also, a floating content approach is used to ensure the availability and survivability of these pieces of information within their relevant areas. It is important to notice that, although NDN solutions use interest-based approaches, no TMS solution employs it for reducing network contentions.

## 3. IAN3I: I Am Not Interested in It

Urban scenario modeling: We represent the road network by a simple directed graph G=(V,E), where *V* is the set of intersections (vertices), while E⊆V×V corresponds to the set of roads (edges), which connects two intersections *u* and *v* such that u,v∈V. Each road uv∈E has an attribute $\tau_{uv}\in \left[0,1\right]$ representing its traffic condition. Let *N* be the set of vehicles on the network with origin *s*, destination *t* and a path *P* linking *s* to *t* such that s≠t and s,t∈V. Table 2 shows the definitions employed by IAN3I.

A key concern when designing a fully-distributed TMS is how to build and deliver to all vehicles a precise city-wide traffic view without producing network contention. To do so, the pieces of information from all vehicles need to be spread through the entire network. However, if it is not done in an efficient way, several transmissions in a short period of time can overload the network, introducing undesired overhead [8]. In such case, data packets will be lost and vehicles will not have a precise traffic view. Consequently, incapacitating them to get a suitable route guidance. To tackle such an issue, IAN3I builds and delivers the traffic view just to vehicles within critical areas, which are classified as follows: static critical areas, have a known location, time, duration and known characteristics, such as frequent traffic hazards, incidents, traffic congestion during rush hours and periodicity; and dynamic critical areas arise in abnormal locations as results of unexpected traffic incidents [23].

To reduce network contentions, IAN3I provides the traffic view within a critical area as a floating content object. Using an opportunistic content sharing approach, it forwards the floating content object just to vehicles actually interested in it, e.g., vehicles that need the traffic view of such a specific critical area. In this way, whenever a vehicle arrives at or goes toward a critical area, it receives the traffic view about such an area, enabling it to discover a better route in case of bottlenecks in its current route. In other words, the interest of a vehicle should be understood as: I am going towards this direction, is there anything that I should know about it?

Critical areas are represented by C⊂E, which are the set of roads within a problematic region. In particular, to set up a dynamic critical area, IAN3I proactively detects whether an unexpected traffic incident occurs. For that, it relies on a plethora of sensors available in each vehicle to provide real-time information and measurements about its behavior and its components. When an unexpected incident occurs, such as traffic accidents, engine damages, airbags activation and etc, the sensors detect these events and then inform IAN3I, which triggers an alert to warn nearby vehicles and defines a critical area dynamically. Thus, when a vehicle receives a warning, it might employ mechanisms to avoid both physical damages and to prevent the emergence of traffic congestion at this location. In the meantime, a floating content object is produced. For the sake of clarity, the optimal shape definition of each critical area is out of the scope of this work. Thus, we used a simple squared shape. However, any other approach for defining each critical area can be trivially incorporated in IAN3I.

For each floating content object, IAN3I attributes an anchor zone (AZ) defined by a radius $r_{a}\in R_{+}^{*}|r_{a}$ and centered at the critical area. Each AZ is responsible for keeping the floating content objects alive within its area, making them available not only to vehicles within it, but also to vehicles approaching it. It is worth noticing that different AZs can have a different radius according to their needs [23,24].

**Figure 1:**
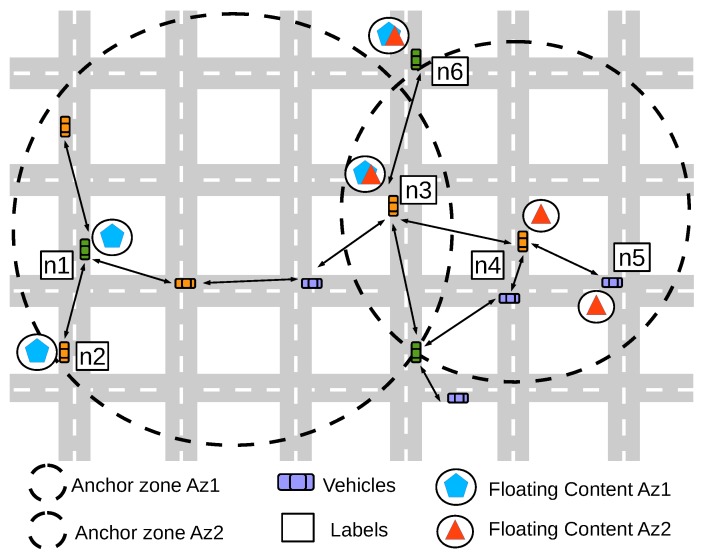
Representation of two different anchor zone (AZ) and their respective floating content objects.

Figure 1 describes the AZs for two floating content objects. In particular, each AZ ($Az_{1}$ and $Az_{2}$) have different sizes and their respective floating content stored. Vehicles $n_{1}$ and $n_{2}$ have the floating content provided by the $Az_{1}$ and vehicles $n_{4}$ and $n_{5}$ have the floating content provided by $Az_{2}$. Therefore, if a vehicle is in an intersection of two or more AZs, it potentially can have the floating content provided from more than one AZ, which is the case of the vehicles $n_{3}$ and $n_{6}$ that have the floating content provided by $Az_{1}$ and $Az_{2}$.

It is important to notice that, at first, IAN3I does not have any kind of knowledge about the traffic condition within the critical areas. Actually, such knowledge is built in a collaborative way and a floating content object is created. Then, it is opportunistically shared and made available to vehicles. Those can then use it to avoid congested roads and unexpected traffic incidents, thus improving their route guidance. Section 3.1 describes how the traffic view of a critical area is built. Section 3.2 describes how IAN3I ensures the availability and survivability of each floating content object. Finally, Section 3.3 describes how vehicles improve their route guidance.

### 3.1. Building Traffic View of Critical Areas

Building an accurate traffic view of a critical area relies on periodic information shared by all vehicles through beacon messages. Each message contains the current position of the vehicle given by its current road $uv_{n}\in E$, its speed s(n), its direction and an identifier that determines if the stored floating content is up to date or not.

Vehicles within a critical area are responsible for sensing the traffic conditions of the roads that belong to it to produce the traffic view. However, to perform such task, there are at least two main issues that needs to addressed: (i) IAN3I must ensure the whole coverage of the critical area; and (ii) IAN3I needs to build an accurate traffic view without introducing an undesired latency to the network.

To tackle the first issue, IAN3I divides the critical area into *k*-smaller sub-areas. The traffic view of each sub-area is built independently. Then, each traffic view is disseminated to all sub-areas to build the floating content object. The number of sub-areas and how they are organized are defined according to the approach proposed in [27], in which the number of sub-areas and their size depend on the communication range of vehicles. In summary, the size of each sub-area is quite close to the communication range of vehicles [27]. On the other hand, to build the traffic view of each sub-area, initially, each vehicle within the sub-area builds its local traffic view by aggregating the beacons received from surrounding vehicles. The local traffic view is represented by L(n), which results in a subset of roads of *C*, corresponding to set the roads within the vehicle’s coverage and their respective average speeds. Upon creating its local knowledge L(n), each vehicle shares it with its neighbors that are in the same sub-area to create the knowledge about the traffic condition of the whole sub-area. A detailed procedure to build such knowledge is described in Algorithm 1.

**Algorithm 1:** Building the traffic view of a sub-area.

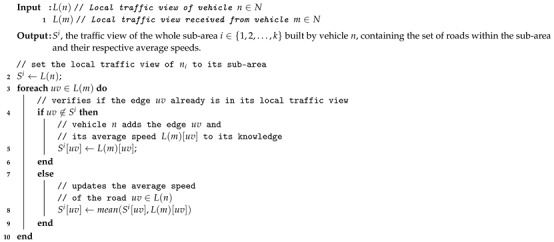



Let $S^{i}$ represent the traffic view of the sub-area i∈{1,2,…,k}. Upon building $S^{i}$, each vehicle computes a traffic condition $\tau_{uv}$ based on the mean speed mens(uv) and max speed maxs(uv) of each road $uv\in S^{i}$ given by:

(1) $$\begin{array}{c} \tau_{uv}=\frac{maxs(uv)-means(uv)}{maxs(uv)}. \end{array}$$

Each vehicle in the same sub-area builds an edge-induced subgraph $G[S^{i}]\subset G$ with the traffic view $S^{i}$, where the set $E\left(G\left[S^{i}\right]\right)\subset E\left(G\right)$ represents the roads within each sub-area, and the set $V\left(G\left[S^{i}\right]\right)\subset V\left(G\right)$ represents the set of vertices that connects each road $uv\in E(G\left[S^{i}\right])$. To build the floating content with the traffic view of the whole critical area, each edge-induced subgraph $G[S^{i}]$ needs to be unified into a single one. Therefore, each subgraph from each sub-area is forwarded to one another. IAN3I employs a broadcast-oriented inter-subarea dissemination, so that it does not overload the network within the critical area. Such decision is based on [8], which employs an efficient broadcast suppression mechanism that reduces the number of packet collisions by cancelling redundant transmissions.

To perform the inter-subarea dissemination, IAN3I selects at least one vehicle in each sub-area to disseminate its edge-induced subgraph $G[S^{i}]$ to the other sub-areas. Since the size of a sub-area is quite close to the communication range of vehicles, IAN3I assumes that all vehicles in the same sub-area potentially have the same edge-induced subgraph. Hence, it may choose any vehicle within a sub-area to disseminate its $G[S^{i}]$. Yet, to perform a better selection, IAN3I chooses a vehicle as close to the center as possible. For that, each vehicle computes its distance to the center of its sub-area. Then, based on such distance, each vehicle schedules a transmission of its $G[S^{i}]$ using a distance-based broadcast protocol which prioritizes vehicles closer to the center (e.g., vehicles closer to the center have shorter delays than others). Moreover, to avoid redundant transmissions if a vehicle overhears the transmission from another vehicle in the same sub-area, it cancels its scheduled transmission. At last, upon transmitting $G[S^{i}]$ of a sub-area, it is forwarded to the other subareas through a multi-hop process, using a broadcast-oriented data dissemination protocol [8].

Due to the inter-subarea dissemination and the division of the critical area into smaller sub-areas, IAN3I is able to maximize the coverage and at the same time reduce the number of transmissions within each sub-area. Notice that, since each sub-area is distinct, a transmission from one sub-area does not cancel the transmission from another one, as they have a different $G[S^{i}]$. However, if such segmentation were not employed, every vehicle would have the same $G[S^{i}]$. Hence, due to the broadcast suppression mechanism, this approach would create gaps in the overall traffic view, since the transmission of a vehicle would potentially cancel the transmission of many others sharing the same region, as they would be considered redundant. However, the information about those vehicles is important to provide precise knowledge about the traffic condition.

With all subgraphs, IAN3I creates a floating content object, which contains a subgraph $G^{\prime }\subseteq G$ representing the whole critical area, given by the Equation 2. Moreover, the floating content object contains an anchor radius $r_{a}$ to determine its geo-location, a timestamp to inform when the floating content was created and an identifier to identify which critical area such floating content object corresponds to

(2) $$\begin{array}{c} G^{\prime }=\overset{k}{\underset{i=0}{⋃}}G\left[S^{i}\right], \end{array}$$

**Figure 2:**
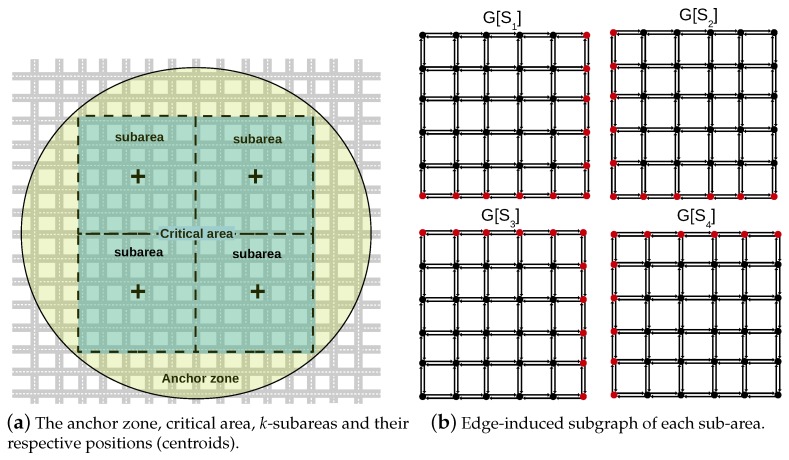
Critical areas, sub-areas, anchor zones and edge-induced subgraphs.

Figure 2 illustrates the main concepts discussed above. In particular, Figure 2a shows an AZ, a critical area, its sub-areas and their respective positions.Moreover, Figure 2b shows the edge-induced subgraphs $G[S_{i}]$ for each sub-area. The red vertices represent the common vertices of two subgraphs, i.e., the vertices that are used to join all subgraphs into a single one to represent the whole critical area.

For the sake of clarity, the survivability and validity of a floating content object depends on its critical area. Thus, in the case of static critical areas, such as an area disturbed by recurrent traffic congestion during rush hours, the floating content needs to be kept alive and up to date during the whole existence of such critical area, and as soon as the traffic condition becomes normal, the critical area and, consequently, the floating content disappears. On the other hand, in case of dynamic critical areas, which arise due to unexpected traffic incidents, the floating content must exist during the whole duration of the incident and disappear as soon as the traffic becomes normal.

### 3.2. Opportunistic Content Sharing

The opportunistic content sharing approach is essential to ensure the floating content survivability on an AZ. It is this process that keeps each floating content active within its AZ, making it available to vehicles in the critical area and to vehicles approaching this area. For that, IAN3I employs another zone called a forwarding zone (FZ), which has a radius of $r_{f}\in R_{+}^{*}|r_{f}>r_{a}$ and is used to send back to the AZ the floating content stored in vehicles leaving the AZ. In other words, when a vehicle moves out from the AZ and it still holds the floating content, then it selects another vehicle that is going toward the AZ to forward the floating content. To avoid unnecessary transmission, when a vehicle storing a floating content leaves its FZ, it discards the floating content. This avoids forwarding the floating content to vehicles that are too far from the critical area, since they may not have interest in it or it may be outdated. Notice that as soon as a vehicle approaches a critical area, it receives the floating content describing such critical area with an up to date information about the traffic condition.

The forwarding process ensures the availability of the floating content within the AZ to vehicles approaching the critical area and it depends on the vehicle’s interests. In summary, such interest depends on whether a vehicle will pass through a critical area or whether it stores any floating content that is outdated. This information is obtained through beacon messages periodically exchanged by vehicles. Therefore, when a vehicle storing an up to date floating content receives a beacon from another vehicle, and they both are within the AZ or FZ, it will forward the floating content based on the probability *p*:

(3) $$\begin{array}{c} p=\left\{\begin{array}{cc} 1 & \;\mathrm{if}\;\exists uv\in P|uv\in E(G^{\prime })\\ \theta (P) & Otherwise \end{array}\right., \end{array}$$

where, θ(P)∈[0,1] is a decreasing function that gives the forwarding probability in respect to the nearest distance to the AZ, defined as:

(4) $$\theta \left(P\right)=\left(\left(r_{f}-r_{a}\right)-\underset{uv\in P,\;u^{\prime }v^{\prime }\in E\left(G^{\prime }\right)}{arg min}dist\left(uv,u^{\prime }v^{\prime }\right)\right)\cdot 0.001.$$

Algorithm 2 describes the whole opportunistic content sharing procedure to ensure the availability and survivability of a floating content object. It starts when vehicle *n* receives an interest message from vehicle *m*. Upon receiving this message, if *n* does not store a floating content object, it just discards the interest message received. On the other hand, it checks whether it is within any zone of its floating content. If it is not within any zone, vehicle *n* deletes the stored floating content object. Otherwise, it verifies whether vehicle *m* stores the same floating content based on the interest message received, and if its true, vehicle *n* discards the interest message received. Otherwise, vehicle *n* computes *p* to decide whether to forward the floating content to vehicle *m* or not. Moreover, vehicle *n* computes a waiting delay to schedule the transmission of the floating content based on the inverse of the distance between vehicles *n* and *m*. In the meantime, to minimize the number of redundant transmissions and to not overload the network, a broadcast suppression mechanism is employed. Therefore, if vehicle *n* detects the transmission of the same floating content currently scheduled, it cancels its own transmission to avoid duplicated messages. In summary, the opportunistic content sharing is defined on whether or not to forward the floating content based on the interest message received from its neighbors and its current location.

Due to the opportunistic content sharing based on floating content, IAN3I does not need to overload the network with repetitive transmissions of the same message to spread a given content to the whole network, thus providing a in-network caching for the traffic view of each critical area. Also, the proposed approach ensures that all vehicles that will pass through a critical area will receive a warning once they approach such area and are interested in the warning. In other words, the content is held close to the area in which it is actually relevant, waiting for vehicles that have an interest on it to arrive on such area to promptly receive the content in a opportunistic way. Such a localized mechanism greatly reduces the network overhead.

**Algorithm 2:** Opportunistic content sharing procedure to ensure the availability and survivability of a floating content.

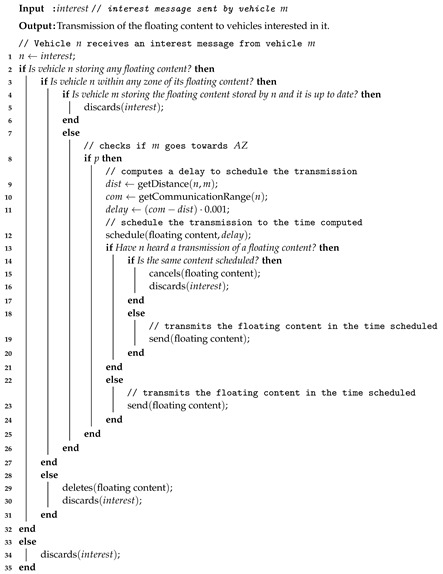



### 3.3. Distributed Traffic Management

The floating content object describing the traffic view of a critical area enables vehicles with such objects to detect congested roads and to know about unexpected traffic incidents. Hence, it allows vehicles to improve their route guidance by computing alternative routes to improve traffic efficiency. Furthermore, the approaches employed by IAN3I provide a distributed traffic management which increases the system scalability, since it avoids the computation burden on a central server. In other words, IAN3I reduces the re-routing algorithm complexity when compared to centralized solutions, consequently providing real-time re-routing.

With the floating content object, each vehicle can detect congested roads based on the traffic condition τuv of roads within a critical area $uv\in E(G^{\prime })$. Each vehicle employs a congestion detection procedure as soon as it receives some floating content object. For detecting traffic congestion, IAN3I relies on the level-of-service (LOS) of the highway capacity manual (HCM) [28]. LOS represents a quality measurement used to describe the percentage of operational conditions within a traffic flow. HCM defines six different levels, with LOS A representing free-flow traffic conditions and LOS F representing high traffic congestion levels. Table 3 shows the relation between the value $\tau_{uv}$ computed by IAN3I and its classification based on the LOSs.

Before re-routing itself, each vehicle needs to check whether such action actually is necessary. In this way, first it verifies whether it is going towards some congested road by analyzing the traffic condition of each road that compose its path. In summary, each vehicle checks if there is any road within its path which has a traffic condition greater than a threshold defined as ε that indicates some congestion. This verification is given by the function σ(P)

(5) $$\begin{array}{c} \sigma \left(P\right)=\left\{\begin{array}{cc} 1 & \exists uv\in P|\tau_{uv}\geq \varepsilon \\ 0 & Otherwise \end{array}\right., \end{array}$$

where the value 1 means that the vehicle has a congested road in its current path, since it has a traffic condition above than the threshold defined according to the LOS classification, and 0 if all roads in its current path have weights bellow the defined congestion threshold.

Vehicles going towards some congested road (e.g., caused by poor mobility or even by unexpected incidents) must be re-routed. Therefore, to avoid creating different congestion points, IAN3I implements a non-deterministic re-routing algorithm based on the Boltzmann probability [29]. The key idea of the algorithm is to first compute a set o *k*-possible alternative paths based on the current position of the vehicle and its destination, then use a probabilistic approach to select one path among the alternatives.

Let P be the set of *k*-possible alternative paths computed using the k-shortest paths algorithm. Also, let *k* and *T* be the two parameters used by the Boltzmann algorithm, in which the first one represents the Boltzmann constant and the other one the parametized attribute. In this way, each vehicle computes its Boltzmann constant according to:

(6) $$\begin{array}{c} k\left(P\right)=\sum_{uv\in P}\exp\left(-\frac{\tau_{uv}}{T}\right), \end{array}$$

where, *P* is a possible alternative path in the set P, $\tau_{uv}$ is the traffic condition of each road in the path and *T* is the parametized value of the Boltzmann algorithm. Notice that, the greater the value of *T*, the higher is the chance of achieving a uniform distribution, which must be avoided in order to provide a better path selection (e.g., avoid selecting either a longer alternative path or a least efficient one).

After computing the Boltzmann constant, each vehicle is able to compute the selection probability of each P∈P based on the traffic condition of the whole path. The probability is defined by Pr(P):

(7) $$\begin{array}{c} Pr\left(P\right)=\frac{1}{\exp\left(\frac{\sum_{uv\in P}\tau_{uv}}{k(P)\cdot T}\right)}, \end{array}$$

where, *P* is a possible alternative path and $\sum_{uv\in P}\tau_{uv}$ represents its traffic condition.

Finally, based on the probability of each path, the vehicle is able to choose its alternative route $P^{\prime }$ using a random procedure based on each probability. The key idea is to select the path with the highest probability that satisfies the following condition:

(8) $$P^{\prime }=\underset{P\;\in \;P}{\mathrm{arg min}}\left\{X\cdot Pr(P),X\in [0,1]\right\},$$

where, *X* is a random variable to represent the random procedure and can assume values between 0 and 1.

It is important to notice that if the critical area does not cover the whole scenario, the aforementioned procedure is applied only for the piece of the path within the critical area. Thus, we first split the current path based on the last road within the critical area to apply such approach, then the remaining roads of the old route are appended to the new one.

## 4. Performance Analysis

This section presents the performance evaluation of IAN3I. Section 4.1 presents the simulation tools, the scenario used and the analyzed metrics. Section 4.2 shows an analysis of IAN3I by studying the impact of the size of the AZ and the size of the critical area. Section 4.3 and Section 4.4 present the network cost and traffic efficiency assessment when dealing with recurrent traffic congestion and unexpected traffic incidents, respectively. For both studies, IAN3I is compared to distributed and hybrid solutions.

### 4.1. Methodology

The simulation platform employed in the performance evaluation of IAN3I is composed by the network simulator OMNeT++ 4.3 [30] combined with the simulation of urban mobility (SUMO) [31] version 0.25, which manages the mobility of vehicles. For the vehicular network, we used the framework Veins 4.3 [32], which implements the IEEE 802.11p and the signal attenuation model that considers the effects of obstacles, such as buildings in urban areas.

To evaluate the solutions in a realistic scenario, a subset of the TAPASCologne 0.17.0 [33] was chosen. TAPASCologne is an open source project that provides a large-scale data set with the highest realism for urban vehicular simulation based on SUMO. It uses a realistic map of the city of Cologne, Germany, obtained from OpenStreetMap [34]. Moreover, using the travel and activity patterns simulation (TAPAS) methodology and the Gawron’s traffic assignment algorithm, it generates a traffic demand from 6:00 a.m. to 8:00 a.m. Section 4.4 describes both scenarios used in our simulations.

As parameters, we set the bitrate to 18 Mbit/s at the MAC layer and the transmission power to 2.2 mW, resulting in a communication range of approximately 300 m under a two-ray ground propagation model. Table 4 summarizes the main simulation parameters used in our assessment.

To evaluate the network cost of all solutions, we assessed the following metrics:

- Delivery ratio: the percentage of messages successfully delivered to vehicles interested in them. It is desired that an efficient TMS delivers about 100% of its generated messages for managing the traffic efficiently.
- Transmitted messages: the total number of messages transmitted by a TMS to guarantee its service. A high number of messages transmitted is an indication that redundant transmissions are occurring.
- Packet delay: the average delay to deliver a message to a vehicle. It is a metric to identify a potential overload of the network. A high delay to receive an information potentially degrades the TMS efficiency when dealing with traffic congestion.
- Packet collisions: the average number of packet collisions in the MAC layer per vehicle to transmit its data messages. It is a measure that indicates how overloaded the network is. In other words, it characterizes the presence of broadcast storm problems due to a high number of message transmissions in a short period of time.

On the other hand, to perform the traffic efficiency evaluation, we analyzed the following metrics:

- Average travel time (ATT): the mean value of the travel time for all vehicles’ trips. It indicates the overall traffic condition for the whole observed scenario.
- Time loss (TL): the mean value of time loss in the travel time for all vehicles’ trips. It indicates the time a vehicle spends stuck in traffic and/or to detour from it.

#### 4.1.1. Recurrent Traffic Congestion Scenario Description

**Figure 3:**
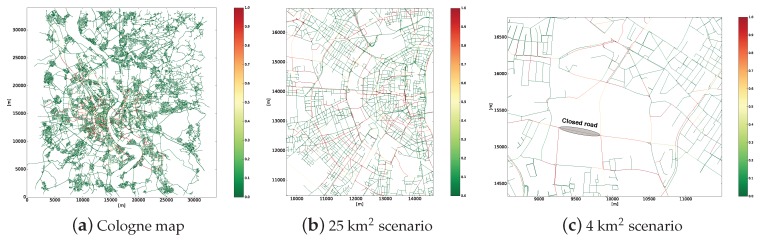
Cologne scenario with its roads colored by traffic condition classified by I am not interested in it (IAN3I) in the rush hour.

In this scenario, we study how the traffic efficiency of some areas degrade due to recurrent traffic congestion such as traffic congestion caused by high density of vehicles (rush hours). In other words, in this scenario, we simulate static critical areas. Figure 3a shows the entire TAPASCologne scenario colored according to the traffic condition at each road during the rush hour. As it can be seen, just some areas are affected by traffic injuries (e.g., critical areas). Therefore, we have selected a subset of ≈25 km^2^ from the TAPASCologne scenario, which contains a critical area with traffic efficiency problems. Figure 3b shows the 25 km^2^ subset selected, presenting the roads colored according to their traffic condition in the rush hour.

#### 4.1.2. Unexpected Traffic Incident Scenario Description

In this scenario, we analyzed how an unexpected traffic incident generates an abnormal traffic condition that disturbs the traffic condition within a specific area. Basically, in this scenario, we simulated dynamic critical areas. To evaluate the impact of an incident in the overall traffic efficiency, a smaller fragment of ≈4 km^2^ from TAPASCologne was chosen. Furthermore, to simulate such an incident, we simulated an accident that completely closes an important road. Figure 3c shows the subset of ≈4 km^2^, the closed road and the roads colored according to the traffic condition. Notice that such traffic condition was a result of the unexpected traffic incident that was added to the scenario.

### 4.2. Anchor Zone and Critical Area Size Evaluation

This section describes how IAN3I behaves according to the size of each AZ as well as to the size of the critical area. With the AZ we study: (i) what is the network cost of the opportunistic content sharing mechanism; and (ii) how is the content availability and survivability within the AZ. On the other hand, regarding the size of the critical area, we study: *(i)* what is the network cost for building a floating content within a critical area.

Table 5 shows the results for the opportunistic content sharing and traffic efficiency analysis according to the size of each AZ. The results show the efficiency of IAN3I in keeping the content alive while providing an efficient in-network caching for the traffic view without inducing high overhead.

The network cost evaluation shows that IAN3I is able to keep an availability greater than 98% for all assessed sizes during the whole duration of a critical area, ensuring the content survivability. Moreover, its interest-based approach avoids redundant transmissions and packet collisions caused by uncontrolled data transmissions. Consequently, it neither overloaded the network nor introduced an undesired latency (see packet collision and delay results). However, as expected, the greater the size of the AZ was the greater was the number of messages transmitted. In particular, a $r_{a}$ of 2.5 km increased the number of messages transmitted in about 40% when compared to a $r_{a}$ of 1.5 km. Despite that, IAN3I was still able to keep a content availability greater than 98%.

The traffic evaluation shows that the accurate traffic view provided to vehicles within or approaching a critical area paves the way to achieve a better traffic management. However, the value of the parameter $r_{a}$ needs to be adjusted properly, not only to provide a good coverage of the critical area, but also to deliver the traffic view quickly, so vehicles can improve their route guidance within appropriate time. It is important to emphasize that the results were obtained using a single critical area with a size of 9 km^2^.

We can see that the $r_{a}=1.5$ km corresponds to an AZ way smaller than the critical area, while the others represent AZs with approximately the same size of the critical area. Thus, $r_{a}=1.5$ delivers the traffic view too late to vehicles, which does not allow them to improve their routes. Conversely, the others deliver the traffic view sooner and with a good coverage of the critical area, thus providing better traffic management. When using $r_{a}$ with an appropriate size, IAN3I decreases the ATT and TL in up to 25% and 51%, respectively. Notice that, $r_{a}$ with values 2.0 and 2.5 km present similar traffic efficiency results. However, regarding the network cost, $r_{a}=2.0$ km decreases the number of messages transmitted in about 15% when compared to $r_{a}=2.5$ km. This result shows that it is better to hold the content closer to its origin rather than spreading it further when considering network cost.

Table 6 shows the network cost and traffic efficiency results for building the traffic view of a critical area according to its size. The results show that the distributed approach employed by IAN3I does not produce network contentions and provide an accurate traffic view to the majority of vehicles within the critical area. In particular, for all assessed sizes, IAN3I presents a delivery ratio greater than 95%. This is due to the fact that IAN3I employs a broadcast suppression mechanism to disseminate the traffic view over the critical area.

The traffic efficiency results show that the smaller critical areas (e.g., 1 and 4 km^2^) do not cover the entire area affected by the high density of vehicles. Thus, the traffic view provided by those areas is insufficient to perform good traffic management. Conversely, the critical area of 9 km^2^ covers the affected area, thus enabling a traffic efficiency improvement. As it can be seen, it minimizes the average ATT and TL in up to 47% and 67% when compared to smaller critical areas.

The best results occur when covering the entire scenario (e.g., critical area with size of 25 km^2^), but, it presents only a slight improvement of about 3% and 8% in the ATT and TL, respectively, when compared to a critical area of 9 km^2^. However, it increases the number of messages transmitted and packet collisions (see network metrics in Table 6). In summary, the critical area of 25 km^2^ transmits two times more messages than the critical area of 9 km^2^ to improve the travel time in just 3%, which is not worthwhile from the network cost point of view.

### 4.3. Network Evaluation

**Figure 4:**
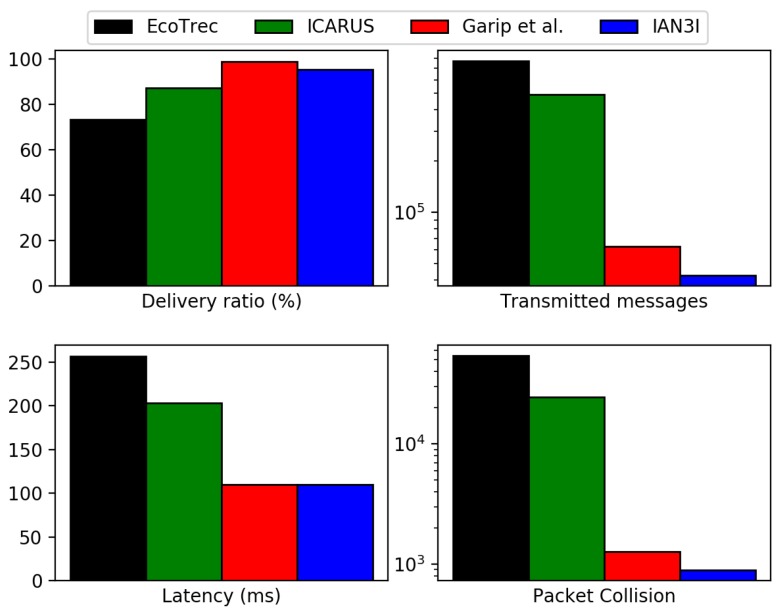
Results of network evaluation metrics for EcoTrec, ICARUS, Garip et al. and IAN3I when dealing with recurrent traffic congestion.

**Figure 5:**
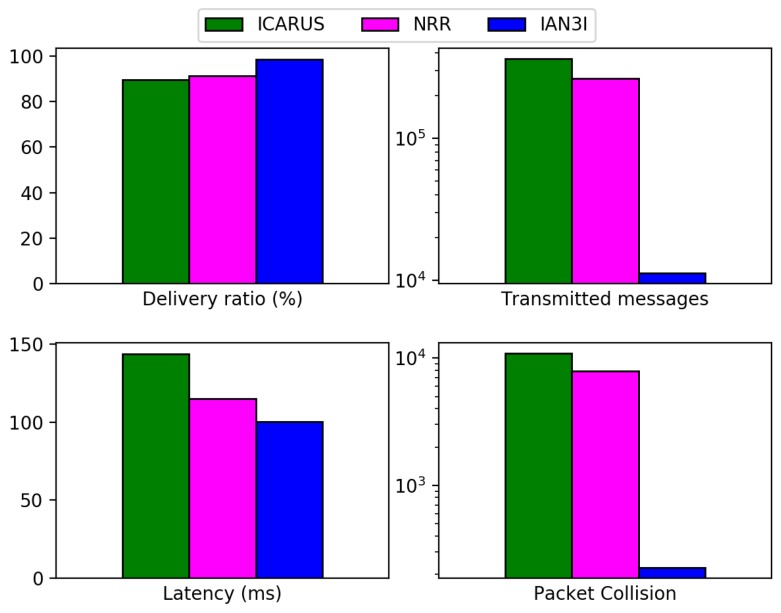
Results of network evaluation metrics for ICARUS, NRR and IAN3I when dealing with unexpected traffic congestion.

We now compare IAN3I with the following literature solutions: EcoTrec [2]; ICARUS [8]; the solution proposed by Garip et al. [17] and NRR [14]. For the sake of clarity, solutions which were not designed for dealing with a specific problem were omitted in the results. Therefore, NRR was omitted from the recurrent traffic congestion scenario evaluation, and EcoTrec and Garip et al. were omitted from the unexpected traffic incident evaluation. Figure 4 shows the results for the evaluated network metrics in the recurrent traffic congestion scenario, while Figure 5 shows the network evaluation results during an unexpected traffic incident scenario.

As shown in Figure 4, hybrid approaches (e.g., which relies on a main server to build a traffic view), such as EcoTrec and ICARUS, increase the network traffic, since all vehicles need to report their traffic-related information at least once per re-routing phase. Thus, it dramatically increases the number of messages transmitted. Due to the lack of mechanisms to either provide an in-network caching or forward information just to vehicles actually interested in it, these approaches induce network contentions by decreasing the delivery ratio and increasing packet collisions and latency.

Conversely, Garip et al. and IAN3I reduce network traffic when compared to hybrid solutions. To do so, Garip et al. uses a request/reply approach based on local information, transmitting a message just when it is requested. IAN3I uses an interest-based approach combined with in-network caching provided by the floating content. This avoids unnecessary and duplicated transmissions. Hence, due to the efficient mechanisms employed by these solutions, they decrease transmissions, packet collisions and latency while increasing the delivery ratio. The decrease is up to 95% in messages transmitted, 98% in packet collisions and 55% in latency when compared to hybrid approaches.

Notice that, Garip et al. transmit more messages than IAN3I because it does not have any in-network caching support. Hence, when many vehicles in the same region request the same pieces of information, this solution transmits as many messages as requested. In other words, it does not avoid duplicated transmissions.

We can observe the same behavior in the unexpected traffic incident scenario (Figure 5), in which ICARUS and NRR do not employ any mechanism to either store traffic incident information close to its location or to forward it just to vehicles interested in it. Instead, they rely on periodic broadcasts to spread the unexpected traffic incident warning. Since IAN3I does employ such approach, it can reduce the messages transmitted, packet collisions and latency by up to 97%, 98% and 30% respectively.

### 4.4. Traffic Efficiency Evaluation

The traffic efficiency evaluation was performed using a relative approach to capture the improvement in travel time and time loss for each vehicle. To do so, the relative result is obtained as the ratio between the result of a specific TMS approach and the results of the original vehicular mobility trace, in which vehicles do not perform re-routing.

**Figure 6:**
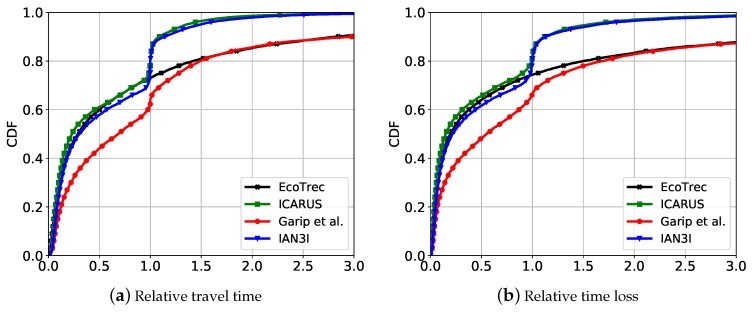
Traffic efficiency results using relative metrics for dealing with recurrent traffic congestion.

**Figure 7:**
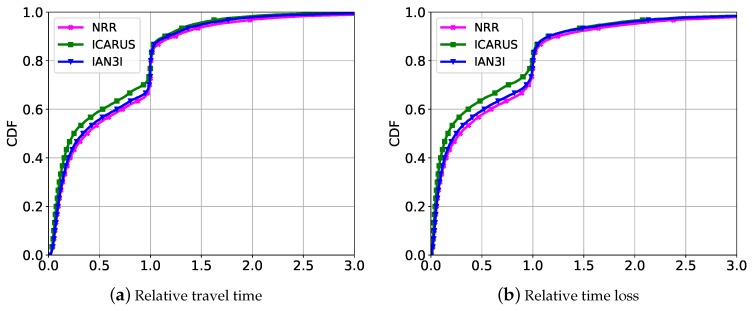
Traffic efficiency results using relative metrics for dealing with unexpected traffic congestion.

Figure 6 and Figure 7 show the traffic efficiency for the relative metrics represented by a cumulative distribution function (CDF) for the recurrent traffic congestion and unexpected traffic incident scenarios, respectively.

Considering the recurrent traffic congestion scenario (Figure 6), the approaches that use deterministic re-routing algorithms are able to improve the mobility for the majority of the vehicles. However, as many vehicles are re-routed toward the same route they create different congestion spots and thus decrease the traffic efficiency of other vehicles. This can be observed when analyzing the results of Garip et al. and EcoTrec. As it can be seen, Garip et al. reduces the travel time and time loss for 60% of the vehicles, while EcoTrec reduces both metrics in 80%. However, they increase the travel time and time loss of impaired vehicles in up to 25 times, which represent 20% of the vehicles in these cases. For the sake of clarity, Garip et al. reduce the travel time and time loss for 20% less vehicles than EcoTrec because it does not have a global traffic view as EcoTrec does.

Since ICARUS and IAN3I use non-deterministic re-routing algorithms, they are able to avoid the limitations presented by Garip et al. and EcoTrec. Hence, they also decrease the travel time and time loss for approximately 80% of the vehicles, of which less than 5% impaired ones. However, for these cases, the increase in the travel time is less than 2 times of the original route.

It is important to notice that, despite its fully-distributed design, IAN3I provides an accurate traffic view of the critical area, enabling vehicles to improve their route, which is a limitation presented by Garip et al. Also, the slight reduction in traffic efficiency when compared to ICARUS is because IAN3I just has the traffic view within the critical area. However, compared to ICARUS, it reduces the complexity of the re-routing algorithm, since it avoids the computation burden in a central server by distributing the route computation to each vehicle, thus increasing the system scalability.

Considering the unexpected traffic incident scenario (Figure 7), we can observe the same behavior. All solutions are able to improve the traffic efficiency for the majority of the vehicles. As expected, IAN3I and NRR reach a performance slightly inferior than ICARUS, since they do not have global traffic view. In particular, IAN3I just has the traffic view of the critical area and NRR the local view of each vehicle.

Finally, with these results we can conclude that: (i) knowing the traffic conditions within critical areas is enough to provide traffic management; (ii) sharing local traffic estimations of an intended area in an distributed efficient way can provide an accurate traffic view of its whole area; (iii) the reduction in data traffic (e.g., number of messages transmitted) provided by IAN3I does not degrade its traffic efficiency at all in both evaluated scenarios; (iv) IAN3I provides an efficient approach for reducing network contention that can be extended to work with other solutions in order to improve their network performance.

## 5. Conclusions

In this work, we proposed IAN3I, an efficient interest-based approach for reducing network contention in vehicular transportation systems. In IAN3I, vehicles are able to cooperatively build an accurate traffic view of affected areas without relying on any kind of fixed infrastructure. Moreover, IAN3I employs an opportunistic content sharing approach in which vehicles within relevant areas are responsible for storing and delivering a traffic view only to vehicles interested in it.

Simulation results in a realistic scenario have shown that, when compared to other solutions, IAN3I improves traffic information delivery, decreases the overhead and packet collisions and induces a low latency while providing an efficient traffic management for dealing with recurrent traffic congestion and unexpected traffic incidents.

As future work, we intend to investigate alternative ways for defining the critical areas, since a rectangular region may not be applicable in some scenarios. Moreover, we intend to extend such approach to other applications to enhance content availability and reduce backhaul traffic.

## Figures and Tables

**Table 1:** Qualitative comparison of I am not interested in it (IAN3I) with literature approaches.

	Architecture			Network Challenges			Traffic View			Routing Strategy		Features				
Related Work	Centralized	Distributed	Hybrid	Broadcast Storm	Network Partitions	In-Network Caching	Global	Critical Areas	Local	Deterministic	Non-Deterministic	Improve Privacy	Interest-Based Forwarding	Request/Reply Based	Periodical Re-Routing	Traffic Incident Awareness
VNS	√						√			√						
Garip et al. [17]		√		√					√		√			√	√	
FOX [13]	√						√				√				√	
Divert [1]			√	√			√				√	√			√	
EcoTrec [2]			√				√			√					√	
NRR [14]			√						√		√					√
ICARUS [8]			√	√	√		√				√				√	√
Navigo [21]		√			√	√										
CODIE [22]		√		√		√										
**IAN3I**		√		√	√	√		√			√		√		√	√

**Table 2:** Summary of definitions employed by I am not interested in it (IAN3I).

Variable	Description
G(V,E)	Road network directed graph
*V*	set of vertices representing the intersections
*E*	set of roads corresponding to the edges, in which E⊆V×V
*C*	C⊆E set of roads within a critical area (e.g., recurrent congested area or area affected by an unexpected traffic incident)
$\tau_{uv}$	traffic condition of the road uv∈E∣u,v∈V
*N*	set of vehicles in the road network
$uv_{n}$	$uv_{n}\subseteq E$ is the current position of the vehicle n∈N given by its current road
*P*	P⊂E is a set of edges representing the route of a vehicle
$S^{i}$	$S^{i}\subset C$ set of roads within a k-subarea, in which i∈{1,2,…,k}
$G[S^{i}]$	$G[S^{i}]\subset G$ edge-induced subgraph representing the subgraph of the i-th subarea
$L^{i}\left(n\right)$	$L^{i}\left(n\right)\subset C$ is the local traffic view built by the vehicle n∈N such that $uv_{n}\in C$
$G^{\prime }$	$G^{\prime }\subseteq G$ subgraph induced by $G[S^{1}⋃S^{2}⋃\ldots ⋃S^{k}]$ all k-subareas

**Table 3:** Relation of $\tau_{uv}$ and its traffic classification.

$\tau_{\mathrm{uv}}$	LOS	Traffic Classification
(0,0.15]	A	Free-flow
(0.15,0.33]	B	Free-flow
(0.33,0.50]	C	Slight congested
(0.50,0.60]	D	Slight congested
(0.60,0.70]	E	Congested
(0.70,1.00]	F	Congested

**Table 4:** Simulation parameters.

Parameters	Values
Channel frequency	5.89e0 Hz mW
Propagation model	Two ray
Transmission power	2.2 mW
Communication range	300 m
Bit rate	18 Mbit/s
Scenario 1	25 km^2^ of Cologne
Scenario 2	4 km^2^ of Cologne
Floating content update frequency	450 s
Critical area size	1, 4, 9, 16, 25 km^2^
$r_{a}$	500, 1000, 1500, 2000, 2500 m
$r_{f}$	500 m larger than AZ
ε	0.5
T	1

**Table 5:** Network and traffic efficiency metrics according to the size of the AZ.

		Anchor Zone Radius $r_{a}$ (km)		
Metrics		1.5	2.0	2.5
**Network**	Delivery ratio (%)	100	99.21	98.78
	Transmitted messages (#)	3997	4554	5402
	Packet delay (ms)	≈100	≈100	≈100
	Collision (%)	1.08	1.11	1.16
**Traffic**	ATT (minutes)	21.23	15.02	15.73
	TL (minutes)	12.16	5.95	5.98

**Table 6:** Network and traffic efficiency metrics according to the size of the critical area.

		Critical Area Size (km^2^)				
Metrics		1	4	9	16	25
**Network**	Delivery ratio (%)	99.27	98.14	97.21	96.05	95.18
	Transmitted messages (#)	279	1034	2563	4572	6997
	Packet delay (ms)	80	100	120	140	150
	Collision (%)	1.97	1.98	2.02	2.03	2.03
**Traffic**	ATT (minutes)	28.42	21.17	15.02	14.69	14.52
	TL (minutes)	19.35	12.68	5.95	5.62	5.45
